# The Hospital

**Published:** 1917-12-01

**Authors:** 


THE
### INSTITUTIONAL WORKER
**Being a Special Supplement to "The Hospital"**

### OUR BUREAU OF INFORMATION.

### Rules for Correspondents.

1. Every letter must be accompanied by the coupon to be cut from the back cover (inside page) of The Hospital, current issue, and must contain the name and address of the correspondent with pseudonym for publication if desired. Replies by post cannot be given save under exceptional circumstances at the Editor's discretion.

2. Letters from Approved Homes in reply to special needs published in the Bureau should state terms and full particulars, and be sent prepaid under cover to the Editor of the Bureau with name written across coupon for identification.

3. Proprietors of Homes which have not yet been entered on the List of Approved Homes, but have spare accommodation likely to suit special needs, are invited to write for an application form for registration. The fee for registration, which includes two announcements of the Home in the Bureau and other privileges, is 10s.

4. All communications to be addressed to the Editor of The Hospital, 28 Southampton Street, Strand, London, W.C. 2, and marked "Bureau of Information."

### Starting an Auxiliary Military Hospital.

### The Staff and Equipment.

School buildings in large towns have been frequently utilised for military hospital purposes. Putting aside the question whether in the ultimate interests of the nation it is desirable to divert these buildings from their original purpose, unless equally suitable accommodation can be found elsewhere for the education of the children whose ejection is thus rendered necessary, school buildings of the best type possess many advantages for temporary hospitals. The more modern are built one storey high, thus being much easier to administer. The class-rooms make splendid wards, the whole place is heated, the sanitary arrangements are usually very good, comprising large lavatories with plenty of space to fix up a bed-pan sluice, and as a rule a sink is fixed, whilst in the cloak-rooms there are plenty of lavatory basins for washing, hot and cold water, and sufficient room to fix up a bath or two. Arrangements should be made, if possible, to have four baths fixed, two at each end of the building. If this is not convenient, there should be one central bath.

The aim should be to fit up these temporary hospitals as cheaply and efficiently as possible. A good deal depends on the nature of the cases admitted. If convalescents only are taken in, a theatre would not be wanted, the nursing staff need not be so large, and fewer surgical appliances and ward equipment would be needed.

We will, therefore, consider the fitting-up of a temporary hospital of 150 beds for acute cases. To work the place efficiently and smoothly, the following staff and equipment would be necessary. It is slightly in excess of the rigid allowance of the military authorities, but it is not possible to work a place well and to do justice to the patients on much less. A great many articles to add to the patients' comfort can be had free, and these are included in the lists, such as bed-tables, bed-rests, self-propelling chairs, easy-chairs, cushions, wheeled couches, small smoking-tables, etc. These can be got from the various societies that supply these articles to military hospitals which cannot always get them in the ordinary way through the military authorities. Linen, bandages, etc., can, as a rule, be had from the local Red Cross Societies. The school authorities leave cupboards, tables, chairs, fire-buckets, etc., and any utensils they may have for cooking. A telephone would have to be installed to ring up the surgeon in case of emergency. It is presumed that the whole staff, both nursing and domestic, would sleep out, but have their meals in the hospital. The surgeons would be visiting and not resident.

### The Nursing Staff.

This should number eleven nurses, six V.A.D.s, and six R.A.M.C. orderlies. For military patients, the nursing staff allowed usually works out at one nurse to ten patients. It is not always easy to adhere to this. Where there are several small wards, an extra nurse has to be put on duty, or in a large ward a nurse may have twelve or fourteen patients.

According to the military scale, a sister is supposed to take charge of from fifty to seventy-five beds. If the hospital is auxiliary to the military hospital in the district, no matron would be appointed, but a sister-in-charge, as the place would be worked under the directions of the principal matron, who would visit the hospital daily. If the hospital be worked independently, a matron would be engaged to take charge of the nursing staff.

The nursing staff would be thus divided:

Day Staff.

- 1 sister-in-charge, (to take theatre when necessary).
- 3 ward sisters (50 beds each).
- 3 staff nurses (one for each sister).
- 3 V.A.D.s.
- 1 N.C.O. R.A.M.C., to be responsible for stores, clothing, etc.
- 3 R.A.M.C. orderlies.

Night Staff.

- 1 night sister-in-charge.
- 3 staff nurses.
- 3 V.A.D.s.
- 2 R.A.M.C. orderlies.

The sister-in-charge is responsible for the domestic staff, the kitchen, and the staff dining-rooms. Her time is also taken up with answering letters, etc.

The R.A.M.C. orderlies do all the portering, keep the outside premises clean, and also assist with the cleaning, especially the corridors.

Domestic Staff.

*Kitchen.*—One cook, one assistant cook, two maids or charwomen, one charwoman to be in the kitchen half-day (morning only), and assist in the cleaning of other departments, such as the dressing-room, during the afternoon, or do relief work.

There should be one staff maid who should be responsible for the cleaning of the sister's office, the nurses' cloak-room (the latter is often used by the nurses as a sitting-room if they do not wish to go out in their off-duty time), and the dining-room. She should be relieved for her half-day by the second maid from the kitchen.

*Ward Cleaners.*—One should be allowed for each sister. This will be sufficient with the help of convalescent patients, who will do the washing-up, clean knives and forks, etc. There would be no fireplaces or staircases to clean. These women have their food with the domestic staff in the kitchen. Ward cleaners are only allowed dinner. A separate dining-room is not usually provided for the kitchen staff unless the kitchen is very small, when a classroom could be fitted up with a table and chairs. Crockery, cutlery, and linen are allowed in the kitchen equipment. It is better, if possible, to get respectable charwomen for the kitchen posts in preference to young maids.

The salaries usually paid to the domestic staff are, the cook £1 1s. a week and her food, the rest of the maids 2s. 6d. a day and their food.

(*To be continued.*)

### Question for December.

**A System of Time-keeping for the Nursing and Domestic Staff.**

What is the most satisfactory method of checking the going out and coming in of the nursing and domestic staffs of an institution which has three outlets, none of which it is practicable to keep locked during the day, nor is it possible to keep porters on duty for the purpose?

### RULES.

The following rules must be observed:—

1. Contributions must be written on one side of the paper. Brevity and terseness are desirable features. The MSS. must bear the name and address of the sender and be accompanied by coupon to be cut from the back cover (inside page) of the current issue of The Hospital. A pseudonym must be chosen if the name is not to be published.
2. Contributions must be addressed to the Editor of The Hospital, Institutional Worker Supplement, 28 & 29 Southampton Street, Strand, London, W.C. 2, should reach him before the end of the current month, and be marked in the left-hand corner "Facts and Figures."

**A minimum payment of Half-a-Guinea will be made for each published answer.**

### QUESTIONS INVITED.

In connection with our Question Box, we offer every month five shillings each for the two best questions which are sent in for consideration. The questions may be on any institutional subject and concern any department, from the secretary's to the porter's, or the matron's to the domestic staff; the one essential is that they must be practical and deal with points that have a definite relation to hospital or institutional administration, upkeep, finance, or management. Points relating to artificers' work, housekeeping, the laundry, out-patients, and other practical matters will be welcome. It is hoped that thus, when difficult or doubtful points arise in the course of their work, institutional workers will be encouraged to put them in the form of a question and send them to the Editor, The Hospital Bureau of Information, 28 Southampton Street, Strand, W.C. 2, marked "Question Box."

The best questions will be published in due course and our readers will be given the opportunity of answering them. Thus all institutional workers may be able to co-operate with the view of helping the work and smoothing the difficulties of each other. In short, we want the Question Box of the *Institutional Worker* to be freely used by every worker habitually.

### ENQUIRIES AND ANSWERS.

### *EMPLOYMENT AND TRAINING.*

### Special Probationers in Hospital for Sick Children.

Two or three vacancies for paying probationers occur yearly at the Evelina Hospital for Sick Children, Southwark Bridge Road, London, S.E. Ladies between the ages of twenty-one and thirty are received after one month's trial for one year's training, and they may, subject to the approval of the Lady Superintendent, continue their training without further payment for another two years. The premium of £13 13s. is payable in advance the first quarter, and after that £1 1s. per week, payable four weeks in advance. Paying probationers must pay their own laundry expenses, except for the washing of uniform, which is provided. No salary is given for the second and third years.—M. N. R.

### Training in Dublin.

Sir Patrick Dun's Hospital, Grand Canal Street, Dublin, is, no doubt, the training-school you refer to. Applicants are received from October to June for four and a half years' training and service. At the end of three years probationers may be required to serve either in the hospital or on the district or private nursing staff. A premium of £25 is asked for, and the salary is £8, £9, and £12 a year respectively. Laundry and indoor uniform are provided. You will find a full list of training-schools in "How to Become a Nurse," published by The Scientific Press, Ltd., 28 and 29 Southampton Street, Strand, W.C. 2, price 2s. 10d. post free.—Coleen.

### Training-Schools in Stockport.

The two recognised training-schools in Stockport are the Stockport Infirmary and the Stepping Hill Poor Law Hospital. The first-named institution receives suitable applicants for three years' training. No premium is required, and a salary of £12 the first year and £15 and £18 the two subsequent years. Laundry and indoor uniform are provided and text-books lent. At the Poor-Law Infirmary, which has attached to it a sanatorium for phthisis, the three years' training qualifies (if the candidate is a certified midwife) for the office of superintendent nurse or head nurse under Article 5 of the Poor-Law Institutions (Nursing) Order of 1913. Lectures are given on physiology, anatomy, medical, surgical, and general nursing, bandaging, cooking, massage, and midwifery. No premium is required, and the salary is £10, £15, and £18 the first, second, and third years respectively.—Lassie.

### *THE SICK AND IN NEED.*

### West of England Sanatorium.

The Royal West of England Sanatorium, Weston-super-Mare, is open all the year round. Open wounds are received if they are healthy. Terms of admission are £1 6s. per fortnight during the months of October to March. Men, women, and children over five years of age are received for two weeks and longer if necessary. Hot and cold sea-water baths are obtainable at the sanatorium.—Nina.

### Aid for Chemist's Widow.

You might possibly obtain aid through the Pharmaceutical Society's Benevolent Fund, 17 Bloomsbury Square, London, W.C. You must apply to the Secretary of the Society at the address given, and at the same time give full particulars of your case.—Widow.

### Jewish Converts' Institution.

The address of the Jewish Converts' Institution is Palestine House, Bodney Road, Hackney, N.E. The object of the institution is to provide a home for young Christian Jews, who are entirely cared for. The age of admission is from seventeen to thirty-five years of age; they are retained for three years.—O. V.

### *MISCELLANEOUS.*

### Disposal of X-ray Negatives.

Yes, it is possible to dispose of your waste negatives, and you can obtain quite a fair price for them. One firm of photographic-glass merchants who is prepared to purchase waste negatives is Messrs. T. Head and Co., of 314 Upper Street, Islington, N., and they guarantee to destroy all films. The prices they offer are 21s. per 1,000 for half-plate size; 50s. per 1,000 for 1 plate; 85s. per 1,000 for $\frac{8}{6}$ plate; 130s. per 1,000 for $\frac{1}{1}\frac{0}{8}$; 185s. per 1,000 for $\frac{1}{1}\frac{5}{2}$.—Hospital Radiographer.

### Tea Supply.

No, a few hospitals experience difficulty in obtaining an adequate supply of tea. The allowance per head should not exceed $1\frac{1}{2}$ oz. per week. We suggest that you apply to the Secretary, Tea Control Committee, 1-3 Oxford Court, Cannon Street, London, E.C.—T. A.

### Railway Vouchers.

The railway vouchers to which nurses, probationers, and masseuses working in hospitals in the jurisdiction of the County Director of the County of London are entitled may be obtained upon application of the matron in charge of hospitals to the County Director. Nurses, probationers, and masseuses must travel in uniform.—Matron.

